# One New Royleanumoate from *Teucrium royleanum* Wall. ex Benth

**DOI:** 10.1155/2014/581629

**Published:** 2014-06-12

**Authors:** Shabir Ahmad, Riaz Ullah, Naser M. AbdElsalam, Hassan Fouad, Ahtaram Bibi, Muhammad Tariq Jan, Anwar Ali Shad, Muhammad Arfan

**Affiliations:** ^1^Department of Chemistry, Islamia College, University Peshawar, Khyber Pakhtunkhwa 25000, Pakistan; ^2^Department of Chemistry, Government College Ara Khel, FR Kohat, Khyber Pakhtunkhwa 26000, Pakistan; ^3^Riyadh Community College, King Saud University, Riyadh 11437, Saudi Arabia; ^4^Department of Chemistry, Kohat University of Science and Technology, Kohat 26000, Pakistan; ^5^Agricultural Chemistry Department, University of Agriculture Peshawar, Khyber Pakhtunkhwa 25000, Pakistan; ^6^Institute of Chemical Sciences, University of Peshawar, Peshawar, Khyber Pakhtunkhwa 25120, Pakistan

## Abstract

One new royleanumoate, a benzene ester (1), has been isolated from *T. royleanum* Wall. ex Benth along with two known compounds, namely, 3,4-dihydroxymethyl benzoate (2) and oleanolic acid (3). The structure elucidation of the isolated compounds was established on two-dimensional (2D) NMR techniques including heteronuclear multiple bond correlation (HMBC), heteronuclear multiple quantum Coherence (HMQC), and correlation spectroscopy (COSY) experiment.

## 1. Introduction

The family Lamiaceae is a large family of order Lamiales [1]. It contains about 170 genera and 300 species of worldwide distribution, growing under great variety of soils and climates but more abundant in Mediterranean and mountainous region [2]. Several genera of the family Lamiaceae contain biologically active compounds [3].* Teucrium* is one of the important genera of this family [4]. The genus* Teucrium* comprises mainly herbaceous plants. It contains about 7,000 species in temperate regions, only four species of which are reported in Pakistan, namely,* Teucrium stocksianum*,* Teucrium scordium, Teucrium royleanum, and Teucrium quadrifarium* [5]. Many biological activitieshave been attributed to the genus* Teucrium*. Some of the species have been used as medicinal plants since time immemorial and are still being used in folk medicine as antispasmodics, tonics, antipyretics, and antiseptics [6]. The literature survey reveals that the terpenoids in these plants have also shown insect antifeedant activity [6–8]. These medicinal properties prompted us to carry out phytochemical investigation on* T. royleanum* in continuation to our ongoing research on this species [9–11]. Our current study has led to the isolation of one new royleanumoate, abenzene ester** 1**. In addition to the new compound** 1**, some known compounds 3,4-dihydroxymethyl benzoate** 2** and oleanolic acid** 3** have been isolated for the first time from this species (see Figures 2, 3, and 4).

## 2. Material and Methods

### 2.1. Plant Materials

The aerial parts of* T. royleanum *were collected from Swat (Pakistan) in June 2003 and identified by Professor Dr. Abdul Rashid, Plant Taxonomist, Department of Botany, University of Peshawar, Peshawar, Pakistan, where a voucher specimen (number Shabir 2651979 (PUP)) is deposited.

### 2.2. Methods for Purification

The powdered air-dried aerial parts of* T. royleanum *(10 kg) were soaked in MeOH (3 × 45 L) at room temperature for about 24 hours. The combined methanolic extract was then concentrated via rotavapour to get a thick gummy extract (850 g). The resultant concentrated extract was then dissolved in water and was subjected to solvent-solvent extraction process using* n*-hexane, chloroform, and* n*-butanol.

The fraction Tb-SA1 was eluted on a silica gel column loaded with initial chloroform-hexane (1 : 1) which on further column chromatography in chloroform-hexane (6.5 : 3.5) provided compound** 1 **as amorphous solid (7 mg).

### 2.3. Physical and Spectral Data of Royleanumoate (**1**)

 IR*ν*_max⁡_ (KBr) cm^−1^ 3440, 1735, 1617
 ElMS* m*/*z*: 121 (100), 107 (6), 71 (11), and 57  (40)
 FAB+MS* m*/*z*: 333.3713 (caled. for C_21_H_34_O_3_)
 
^1^H-NMR ^13^C-NMR (C_5_D_5_N, 400 MHz and 100 MHz): Table 1.

## 3. Results and Discussion

**Scheme 1:**
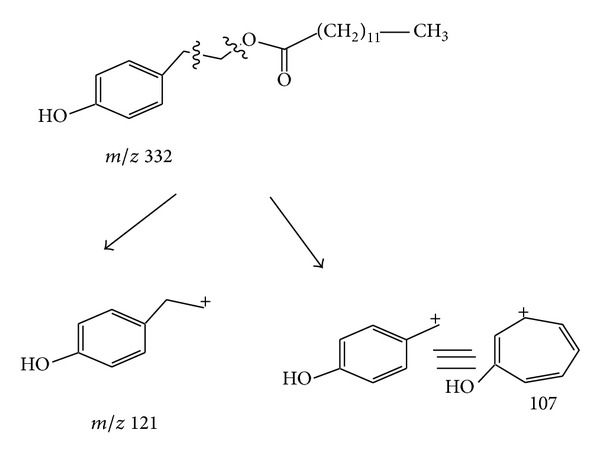
The mass spectral fragmentation pattern for royleanumoate (**1**).

Compound** 1 **was isolated from the VLC fraction of the chloroform soluble part obtained from the methanol extract of* T. royleanum* Wall. ex Benth as amorphous solid. The Fab +ve of** 1 **showed the [M+1]^+^ at* m/z* 333.3713 in agreement with the molecular formula C_21_H_34_O_3_ indicating five degrees of unsaturation. Other prominent mass fragments at* m/z* 121 (100), 107 (6), 71 (11), and 57 (40) were also observed in the mass spectrum as shown in Scheme 1. The IR spectrum of compound** 1 **exhibited absorption bands at 1735 (ester C=O), 3430 for (OH), and 1617 for (aryl).

The ^1^H-NMR spectrum corroborated the presence of one methyl, thirteen methylene, and aromatic groups in the high-field region. In the downfield region of the spectrum two doublets at *δ* 6.75 and 7.05 each of two protons integration were assigned to C-2′, C-6′ and C-3′, C-5′ aromatic protons. The methyl group attached at the terminal position of the aliphatic chain appeared as a triplet at *δ* 0.88 with a* J* = 6.36. Similarly, methylene protons at C-1′′ and C-2′′ at *δ* 2.81 and 2.24 show two triplets each of 2 H integration with a* J* value of 2.81 Hz and 2.25 Hz.

The ^13^C-NMR spectrum (BB, DEPT) (Table 1) showed twenty-one signals, including one methyl, thirteen methylene, four methine, and three quaternary carbons. In the downfield region signals appeared at *δ* 130.2, 115.4, 130.04, and 153.8 which were assigned to the C-1′, C-2′, C-6′, C-3′, C-5′, and C-4′ of aromatic carbons, while a signal at *δ* 173.7 indicated the presence of a carbonyl carbon in the form of ester in the molecule.

Similarly, two signals at *δ* 34.4 and 64.9 were assigned to the methylene carbons present in between ether oxygen and aromatic ring, while in the upfield region a signal at *δ* 14.1 was assigned to the methyl carbon attached at terminal position of the aliphatic chain. The long-range ^1^H-^13^C connectivities were established through HMBC technique.

**Figure 1:**
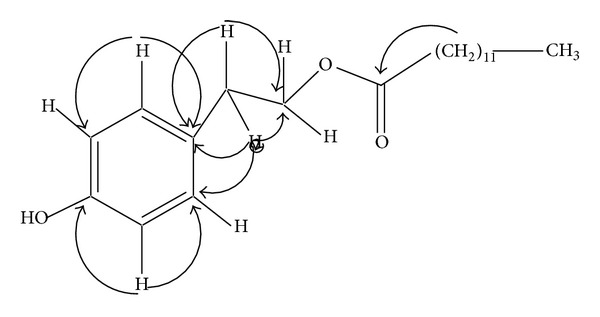
Important HMBC correlations of royleanumoate (**1**).

**Figure 2:**
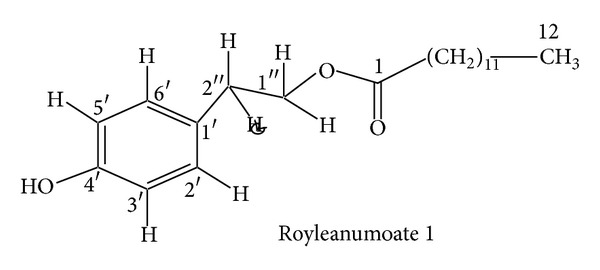


**Figure 3:**
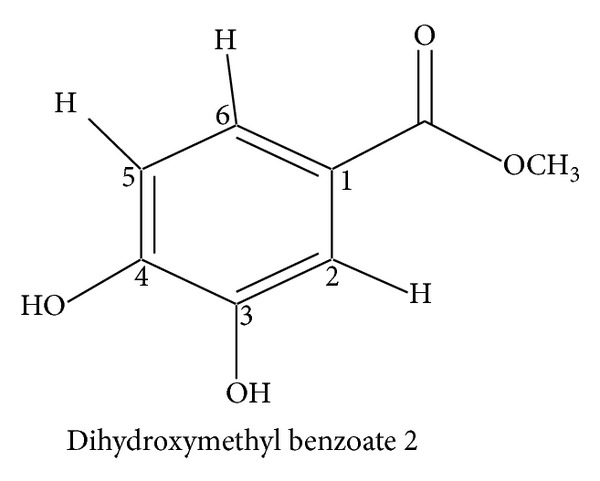


**Figure 4:**
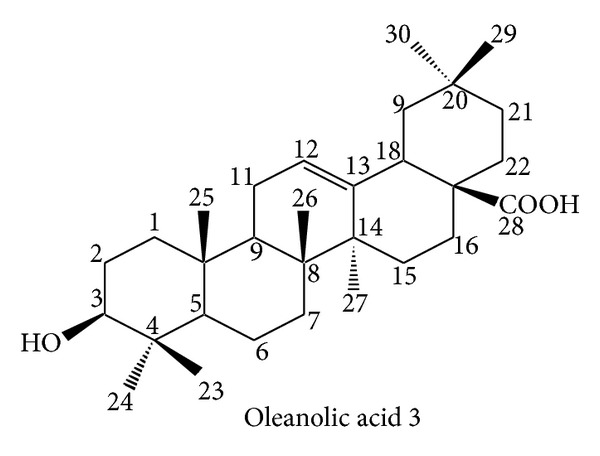


In the HMBC spectrum (Figure 1), the C-1′′ methylene protons (*δ* 2.81, t) showed correlations with C-2′′ (*δ* 34.38) and another correlations of C-2′′ (*δ* 130.04) and C-1′ (*δ* 130.15), thus supporting the attachment of –CH_2_–CH_2_– to the phenol ring at paraposition. Similarly the two orthoprotons (C-2′, C-6′) also showed correlations with C-3′ and C-5′, respectively.

On the basis of all the above spectral data and comparison with the analogous structures in the literature [12] the compound** 1** was named as royleanumoate. 3,4-Dihydroxymethyl benzoate** 2** and oleanolic acid** 3 **were also isolated for the first time from thechloroform soluble fraction of the crude extract of* T. royleanum* and identified by comparison with the literature data [13].

## 4. Conclusion

One new compound (benzene ester** 1**) and two known compounds (3,4-dihydroxymethyl benzoate** 2** and oleanolic acid** 3**) have been isolated from* T. royleanum* Wall. ex Benth. The isolated compounds were confirmed by two-dimensional NMR technique, IR, and mass spectra.

## Figures and Tables

**Table 1:** ^
1^H-NMR and ^13^C-NMR (C_5_D_5_N, 400 MHz, C_5_D_5_N, 100 MHz), chemical shifts, and multiplicities of (1).

C. number	Multiplicity (DEPT)	^ 13^C-NMR (*δ*)	^ 1^H-NMR (*δ*)	^ 1^ *J*_HH_ (Hz)
C-1	C	173.80	—	—
C-2-C-11	CH_2_	29.25	1.23	brs
C-12	CH_3_	14.11	0.88	t, *J* = 6.4
C-1′	C	130.15	—	—
C-3′-5′	CH	115.38	6.75	d, *J* = 10.0
C-2′-6′	CH	130.04	7.05	d, *J* = 10.0
C-4′	C	153.84	—	—
C-1′′	CH_2_	34.38	2.81	t, *J* = 2.8
C-2′′	CH_2_	64.87	2.24	t, *J* = 2.3
